# GlycA, a Pro-Inflammatory Glycoprotein Biomarker, and Incident Cardiovascular Disease: Relationship with C-Reactive Protein and Renal Function

**DOI:** 10.1371/journal.pone.0139057

**Published:** 2015-09-23

**Authors:** Eke G. Gruppen, Ineke J. Riphagen, Margery A. Connelly, James D. Otvos, Stephan J. L. Bakker, Robin P. F. Dullaart

**Affiliations:** 1 Department of Nephrology, University of Groningen and University Medical Center Groningen, Groningen, The Netherlands; 2 Department of Endocrinology, University of Groningen and University Medical Center Groningen, Groningen, The Netherlands; 3 LabCorp, Raleigh, North Carolina, United States of America; The University of Tokyo, JAPAN

## Abstract

**Objective:**

GlycA is a novel nuclear magnetic resonance spectroscopy-measured biomarker of systemic inflammation. We determined whether GlycA is associated with incident cardiovascular disease (CVD) in men and women, examined whether this association with CVD is modified by renal function, and compared this association with high sensitivity C-reactive protein (hsCRP).

**Research design and methods:**

A prospective cohort study was performed among 4,759 subjects (PREVEND study) without a history of CVD and cancer. Incident CVD was defined as the combined endpoint of cardiovascular morbidity and mortality. Cox regression analyses were used to examine associations of baseline GlycA and hsCRP with CVD.

**Results:**

298 first CVD events occurred during a median follow-up of 8.5 years. After adjustment for clinical and lipid measures the hazard ratio (HR) for CVD risk in the highest GlycA quartile was 1.58 (95% CI, 1.05–2.37, P for trend = 0.004). This association was similar after further adjustment for renal function (estimated glomerular filtration rate and urinary albumin excretion). After additional adjustment for hsCRP, GlycA was still associated with incident CVD (HR: 1.16 per SD change (95% CI, 1.01–1.33), P = 0.04). Similar results were obtained for hsCRP (HR per SD change after adjustment for GlycA: 1.17 (95% CI 1.17 (95% CI, 1.01–3.60), P = 0.04). CVD risk was highest in subjects with simultaneously higher GlycA and hsCRP (fully adjusted HR: 1.79 (95% CI, 1.31–2.46), P<0.001).

**Conclusion:**

GlycA is associated with CVD risk in men and women, independent of renal function. The association of GlycA with incident CVD is as strong as that of hsCRP.

## Introduction

It is increasingly recognized that protein glycosylation, i.e. the enzymatic process whereby a glycan (polysaccharide) moiety is added to a protein, affects many physiological processes including the innate immune system, thereby modulating inflammatory responses [1–3]. Circulating glycosylated acute phase proteins are elevated in various inflammatory and autoimmune disorders [4]. It is well known that atherosclerotic cardiovascular disease (CVD) is featured by enhanced low grade inflammation [5,6]. To date, numerous studies have shown that higher circulating levels of acute phase proteins, in particular high sensitivity C-reactive protein (hsCRP), predict future development of cardiovascular disease (CVD) [4,5,7,8], although the possibility that CRP may play a causal role in derangements of inflammatory processes involved in the pathogenesis of atherosclerosis has been questioned [9,10].

GlycA is a recently developed nuclear mass resonance (NMR) spectroscopy-derived biomarker of systemic inflammation [11,12]. This NMR signal arises from the *N*-acetyl methyl groups of the *N*-acetylglucosamine residues located on specific glycan branches of circulating plasma proteins, mainly α1-acid glycoprotein (oromucosoid), haptoglobin, α1-antitrypsin, α1-antichymotrypsin and transferrin. It has been established that GlycA is strongly correlated with hs-CRP, which supports the contention that GlycA is a marker of low-grade systemic inflammation [11,13].

Interestingly, it has been recently shown that plasma GlycA is independently associated with incident CVD in a large cohort study of initially healthy women [13]. In this report, the association of GlycA with incident CVD was similar to that of hsCRP. Of note, the associations of GlycA and hsCRP with incident CVD were attenuated after mutual adjustment for these inflammatory markers, which raises the possibility that GlycA and hsCRP are biomarkers that reflect in part common processes involved in atherosclerosis development. At present it is unknown whether the association of GlycA with future development of clinically manifest CVD also holds true for men. Of further importance, no data are available with respect to the relationship of GlycA with renal function and albuminuria. It is important to determine these relationships, because both lower estimated glomerular filtration rate (eGFR) and higher degrees of albuminuria confer increased risk of cardiovascular morbidity and mortality [14–16]. Moreover, low grade chronic inflammation as inferred from higher hsCRP predicts a decline in renal function, and associates with albuminuria [14,17,18].

We therefore initiated the present study to determine i) whether GlycA associates with increased CVD risk in both men and women, ii) the extent to which the anticipated association of GlycA with future CVD is modified by renal function abnormalities, as inferred from eGFR and albuminuria and iii) the extent to which the anticipated association of GlycA with future CVD is attenuated by hsCRP, representing a widely used marker of low-grade chronic inflammation. To this end a prospective evaluation was performed among participants of the Prevention of Renal and Vascular ENd-Stage Disease (PREVEND) study, a prospective investigation of albuminuria, renal and cardiovascular disease in a large, predominantly Caucasian population.

## Materials and Methods

### Study design and population

Details of the PREVEND study are described elsewhere [19,20]. In summary, in 1997 through 1998, all inhabitants of the city of Groningen, The Netherlands, between the ages of 28 and 75 years (85,421 subjects) were asked to send in a morning urine sample and to fill out a short questionnaire. Pregnant women and subjects with type 1 diabetes mellitus were excluded. The urinary albumin concentration was assessed in 40,856 responders. Subjects with a urinary albumin concentration ≥10 mg/L (n = 7,768) were invited to participate, of whom 6,000 were enrolled. In addition, a randomly selected group with a urinary albumin concentration of <10 mg/L (n = 3,394) was invited to participate in the cohort, of whom were 2,592 were enrolled. These 8,592 individuals constitute the PREVEND cohort. The second screening took place from 2001 through 2003 (n = 6,894), which was the starting point of the present evaluation. The PREVEND study has been approved by the Medical Ethics Committee of the University Medical Center Groningen, and is performed in accordance with Declaration of Helsinki guidelines. All participants provided written informed consent. GlycA and hsCRP were measured in 5,526 subjects in whom previously unthawed samples were available. For the present study subjects with a history of CVD (n = 349) and cancer (n = 418) at baseline were excluded, leaving 4,759 subjects for the analysis.

### Follow-up and outcome

Follow-up time was defined as the period between assessment at the second screening round (baseline) and first CVD event, loss to follow-up, or the end of follow up time (01-01-2011), whichever came first. If a person had moved to an unknown destination, the date on which the person was dropped from the municipal registry was used as the census date.

Data on mortality were obtained from the municipal register, and the cause of death was obtained by linking the number of the death certificate to the primary cause of death as coded by a physician from the Central Bureau of Statistics. Information for cardiovascular morbidity was obtained from PRISMANT, the Dutch national registry of hospital discharge diagnoses. All data were coded according to

the International Classification of Diseases, the Ninth Revision (ICD-9) was used for data until 01-01-2009, after this date, data were coded according to the Tenth Revision (ICD-10). CVD was defined as the combined endpoint of incident cardiovascular morbidity and mortality which includes the following events: acute myocardial infarction, acute and subacute ischaemic heart disease, occlusion or stenosis of the precerebral or cerebral arteries or the following procedures: coronary artery bypass grafting, percutaneous transluminal coronary angioplasty or other vascular interventions (i.e. percutaneous transluminal angioplasty or bypass grafting of the aorta and peripheral vessels). Cardiac events were defined as fatal ⁄nonfatal myocardial infarction, ischemic heart disease, coronary artery bypass grafting and percutaneous transluminal coronary angioplasty.

### Baseline measurements and definitions

Body mass index (BMI) was calculated as weight (kg) divided by height squared (meter). Smoking status was categorized as never, former and current. Alcohol intake was categorized as almost never, 1–4 drinks per month or 2–7 drinks per week, and ≥1 drink per day. Blood pressure was measured with an automatic Dinamap XL Model 9300 series device (Johnson-Johnson Medical, Tampa, FL, USA). Hypertension was defined as a systolic blood pressure (SBP) >140 mmHg or a diastolic blood pressure (DPB) >90 mmHg, or the use of blood pressure-lowering drugs. Type 2 diabetes mellitus (T2DM) was defined as a fasting serum glucose level >7.0 mmol/L, a non-fasting plasma glucose level >11.1 mmol/L, self-report of a physician diagnosis or the use of glucose lowering drugs, retrieved from a central pharmacy registry. eGFR was calculated using the combined creatinine cystatin C-based Chronic Kidney Disease Epidemiology Collaboration equation from 2012 [21].

### Laboratory measurements

Fasting blood samples were provided and stored at -80°C. NMR spectra were collected from EDTA plasma samples using the Vantera^®^ Clinical Analyzer [22]. The GlycA NMR signal is derived from the *N*-acetyl methyl protons of *N*-aceylated carbohydrate side chains of serum glycoproteins (predominantly, α1-acid glycoprotein, haptoglobin, α1-antitrypsin, α1-antichymotrypsin and transferrin) [11]. The GlycA NMR signal is centered at 2.00 ± 0.01 ppm in the NMR spectra of plasma, and only *N*-acetylglucosamine with specific glycosidic linkage, namely, β (1>2) or β (1>6) with a preceding mannose residue, contribute to the GlycA signal [11].

hsCRP was measured by nephelometry with a threshold of 0.18 mg/L (BNII, Dade Behring). Plasma glucose was measured as described [6]. Serum total cholesterol was assayed on an automatic analyser type MEGA (Merck, Darmstadt, Germany) using the CHOD-PAP-method. Triglycerides (TG) and high density lipoprotein cholesterol (HDL-C) were measured on a Beckman Coulter AU Analyzer. Non-HDL cholesterol was calculated as the difference between total cholesterol and HDL cholesterol. Measurement of serum creatinine was performed by an enzymatic method on a RocheModular analyzer (Roche Diagnostics, Mannheim, Germany). Serum cystatin C concentrations were measured by Gentian Cystatin C Immunoassay (Gentian AS, Moss, Norway) on a Modular analyzer (Roche Diagnostics). Urinary albumin concentration was measured by nephelometry with a threshold of 2.3 mg/l, and intra- and inter-assay coefficients of variation of 2.2% and 2.6%, respectively, (Dade Behring Diagnostic, Marburg, Germany).

### Statistical analysis

Statistical analyses were performed using statistical software SPSS version 22.0 (SPSS Inc, Chicago, IL) and STATA version 13.1 (StataCorp, College Station, TX: StataCorp LP). Normally distributed data were expressed as mean ± SD and skewed data as median [interquartile range]. Subject characteristics and laboratory variables were calculated across sex-stratified quartiles of GlycA. P-values across quartiles of GlycA were determined by linear regression for continuous variables or chi-square test for categorical variables. Skewed data were normalized by logarithmic transformation before analyses, which was the case for triglycerides, UAE and hsCRP. Univariable linear regression analyses are presented as standardized beta with corresponding P-value.

Kaplan-Meier curves with log-rank tests were used to estimate survival curves of GlycA and hsCRP levels, either separately or joint, on CVD outcome. Cox proportional hazards regression analyses were used to determine the risk for incident CVD events according to quartiles of GlycA and hsCRP, as well as per 1 SD increase of GlycA and log hsCRP. The assumption of proportional hazards for baseline predictors was investigated by inspecting the Schoenfeld residuals. Multivariable analyses were conducted using Cox regression models including the covariates age, sex, BMI, alcohol intake, smoking status, prevalent T2DM, use of lipid lowering drugs, use of anti-hypertensive medication, SBP, total cholesterol, HDL cholesterol, triglycerides, eGFR, and UAE. Tests of trend across quartiles were conducted by assigning the median value for each quartile as its value and treating this as a continuous variable. Additionally, we also evaluated the association between GlycA and hsCRP and cardiac events only, reasoning the numbers of cerebrovascular and peripheral vascular endpoints were too low to allow for a meaningful subanalysis. Possible effect modification was explored by including the interaction terms between GlycA or hsCRP and age or sex in the multivariable adjusted models.

The additional value of GlycA and hsCRP for CVD risk prediction was assessed by discrimination using Harrell’s C-statistic. The theoretical maximum of 1.0 indicates perfect prediction and a value of 0.5 indicates that patients are correctly classified in 50% of subjects (no discrimination) [23].

The joint associations of GlycA and hsCRP on outcome were evaluated by dichotomizing the distribution of GlycA and hsCRP, according to cut points of the highest quartile of GlycA (>387 μmol/L) and the highest quartile of hsCRP (>2.95 mg/L) in both sexes combined, to test if both biomarkers in the highest range had a different association with first CVD event vs. both these biomarkers in the lowest range or one of these biomarkers in the lowest range.

Given the enrichment of subjects with elevated urinary albumin excretion in the PREVEND population, we also performed a secondary analysis in which we accounted for the sampling design of the study, with respect to enrichment of subjects with a urinary albumin concentration >10 mg/L, by specifying stratum-specific baseline hazard functions.

Interaction terms were considered to be statistically significant at two-sided P-values<0.10 [24]. Otherwise, the levels of significance was set at two-sided P-values <0.05.

## Results

The baseline characteristics of the 4,759 subjects according to sex-stratified quartiles of GlycA are presented in **Table 1**. The mean age of the study population was 52.7 ± 11.8 years. Subjects in the highest quartile of GlycA were older and smoked more frequently compared to subjects in the lowest GlycA quartile. Hypertension and T2DM were more prevalent among subjects in the highest quartile. Levels of hsCRP, total cholesterol, non-HDL cholesterol, triglycerides and UAE were higher, whereas HDL cholesterol and eGFR were lower in subjects in the highest quartile vs. subjects in the lowest GlycA quartile.

**Table 1:** Baseline characteristics according to sex-stratified quartiles of GlycA concentrations in 4,759 participants of the PREVEND study.

	Quartiles of GlycA, μmol/L				
	1	2	3	4	
	♂≤301	♂302–335	♂336–377	♂≥378	P-value
	♀≤311	♀312–350	♀351–392	♀≥393	
Participants, n	1186	1182	1205	1186	
Age, years	48.6±10.6	52.2±11.8	54.5±11.9	55.5±11.8	<0.001
Female, n (%)	641 (54.0)	642 (54.3)	657 (54.5)	641 (54.0)	0.99
BMI, kg/m^2^	24.6±3.4	26.1±3.7	27.2±4.1	28.3±4.9	<0.001
Smoking, n (%)					<0.001
Never	480 (40.4)	376 (31.8)	316 (26.2)	287 (24.2)	
Former	490 (41.3)	519 (43.9)	514 (42.7)	446 (37.6)	
Current	201 (16.9)	273 (20.1)	360 (29.9)	441 (37.2)	
Alcohol intake, n (%)					<0.001
Almost never	222 (18.7)	243 (20.6)	321 (26.6)	363 (30.6)	
1–4 drinks per month	190 (16.0)	214 (18.1)	202 (16.8)	191 (16.1)	
2–7 drinks per week	454 (38.3)	391 (33.1)	363 (30.1)	323 (27.2)	
≥1 drinks per day	310 (26.1)	321 (27.2)	309 (25.6)	299 (25.2)	
Hypertension, n (%)	177 (14.9)	296 (25.0)	436 (36.2)	507 (42.7)	<0.001
Lipid lowering drug use, n (%)	40 (3.4)	58 (4.9)	103 (8.5)	128 (10.8)	<0.001
SBP, mmHg	118.4±15.3	123.8±18.2	127.8±19.7	130.6±18.9	<0.001
DBP, mmHg	70.2±8.6	72.4±8.8	74.0±9.0	74.7±8.7	<0.001
T2DM, n (%)	21 (1.8)	38 (3.2)	74 (61)	106 (8.9)	<0.001
Blood pressure-lowering drug use, n (%)	102 (8.6)	170 (14.4)	277 (23.0)	333 (28.1)	<0.001
Use of glucose-lowering drugs, n (%)	6 (0.5)	19 (1.6)	40 (3.3)	61 (5.3)	<0.001
hsCRP, mg/L	0.52 [0.26–0.95]	0.98 [0.56–1.70]	1.75 [0.93–3.19]	3.61 [1.86–7.10]	<0.001
Glucose, mmol/L	4.7±0.9	4.9±0.9	5.1±1.1	5.3±1.3	<0.001
Total cholesterol, mmol/L	5.2±1.0	5.4±1.0	5.6±1.0	5.7±1.1	<0.001
Non-HDL cholesterol, mmol/L	3.8±1.0	4.1±1.0	4.3±1.0	4.5±1.1	<0.001
HDL cholesterol, mmol/L	1.4±0.3	1.3±0.3	1.2±0.3	1.3±0.3	<0.001
Triglycerides, mmol/L	0.84 [0.64–1.70]	1.03 [0.78–1.97]	1.22 [0.88–1.70]	1.39 [1.02–1.86]	<0.001
eGFR*crea-cysC*, ml/min per 1.73 m^2^	98.2±13.9	93.6±16.4	90.7±16.3	88.0±18.1	<0.001
eGFR*crea-cysC*, ml/min per 1.73 m^2^, categorical					<0.001
≥90 ml/min per 1.73 m^2^	845 (71.2)	689 (58.3)	631 (52.4)	541 (45.6)	
≥60 ml/min per 1.73 m^2^	276 (23.3)	383 (32.4)	450 (37.3)	471 (39.7)	
<60 ml/min per 1.73 m^2^	7 (0.6)	24 (2.0)	43 (3.6)	75 (6.3)	
UAE, mg/ 24h	7.1 [5.7–10.3]	7.6 [5.8–11.4]	8.4 [6.1–13.3]	8.9 [6.1–17.9]	<0.001
UAE, mg/24H, categorical					<0.001
≥30	40 (3.4)	80 (6.8)	109 (9.0)	186 (15.7)	
<30	1141 (96.2)	1096 (92.7)	1090 (90.5)	996 (84.0)	

Data are expressed as mean ± SD, median [IQR] or in number (n) and %. P-values are calculated by linear regression analysis or χ^2^ analysis. Data with respect to smoking and alcohol consumption are missing in 56 (1.2%) and 43 (0.9%) of the subjects, respectively. Abbreviations: *BMI*, body mass index; eGFR*crea-cysC*, estimated glomerular filtration rate based on creatinine-cystatin C equation; *DBP*, diastolic blood pressure; *SBP*, systolic blood pressure; *T2DM*, type 2 diabetes mellitus; *HDL-cholesterol*, high density lipoprotein cholesterol; *hsCRP*; high sensitive- C-reactive protein; *UAE*, urinary albumin excretion; *PREVEND*, Prevention of REnal and Vascular ENd-stage Disease.

**Fig 1:**
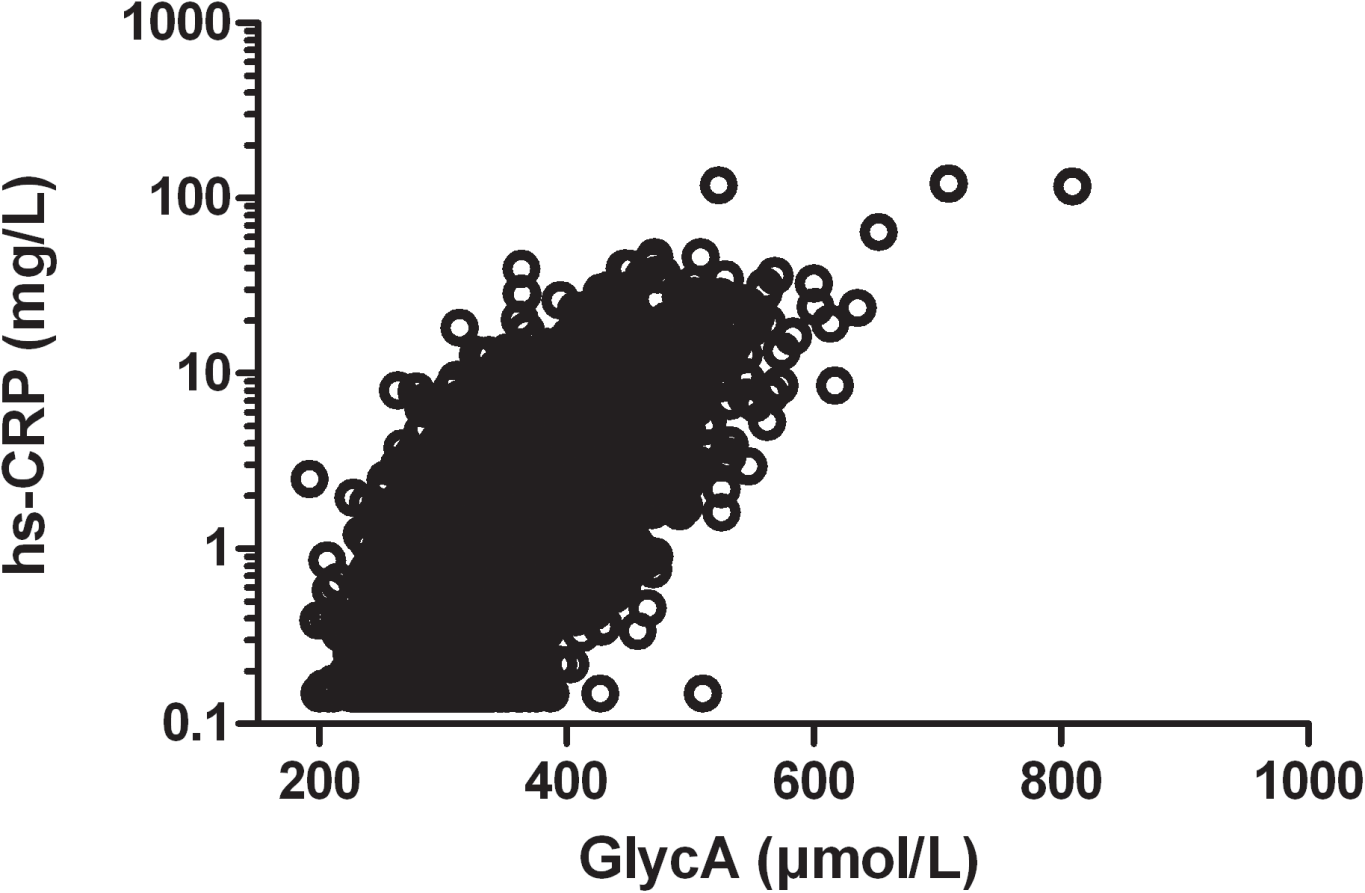
Scatter plot showing the correlation between GlycA and high sensitivity C-reactive protein (hsCRP) (univariate correlation coefficient: 0.67).

The relationship between GlycA and hsCRP is shown in **Fig 1**. Both GlycA and hs-CRP were higher in women than in men (Table 2). Univariable linear regression analyses showed that age, BMI, smoking, hsCRP, glucose, total cholesterol, non-HDL cholesterol, triglycerides and UEA were positively associated with GlycA, whereas alcohol intake, HDL cholesterol and eGFR were inversely associated with GlycA levels (**Table 2**). GlycA was higher in subjects using anti-hypertensive drugs, lipid lowering drugs and glucose lowering medication. Similar associations were observed for hsCRP.

**Table 2:** Univariate associations of clinical parameters with GlycA and hsCRP.

	GlycA		hsCRP	
Clinical parameter	Std B	P-value	Std B	P-value
Age	0.22	<0.001	0.25	<0.001
Sex				
Males	*Reference*		*Reference*	
Females	0.10	<0.001	0.04	<0.001
BMI, kg/m^2^	0.31	<0.001	0.39	<0.001
Smoking				
Never	*Reference*		*Reference*	
Former	0.06	0.001	0.08	0.001
Current	0.19	<0.001	0.12	<0.001
Alcohol intake				
Almost never	*Reference*		*Reference*	
1–4 drinks per month	-0.09	<0.001	-0.07	<0.001
2–7 drinks per week	-0.16	<0.001	-0.12	<0.001
≥1 drinks per day	-0.13	<0.001	-0.09	<0.001
SBP, mmHG	0.22	<0.001	0.23	<0.001
DBP, mmHg	0.16	<0.001	0.18	<0.001
Hypertension	0.23	<0.001	0.23	<0.001
Type 2 diabetes mellitus	0.13	<0.001	0.12	<0.001
Use of blood pressure lowering drugs				
No	*Reference*		*Reference*	
Yes	0.20	<0.001	0.20	<0.001
Use of lipid lowering drugs				
No	*Reference*		*Reference*	
Yes	0.12	<0.001	0.06	<0.001
Use of glucose lowering drugs				
No	*Reference*		*Reference*	
Yes	0.12	<0.001	0.09	<0.001
hsCRP, mg/L	0.67	<0.001	-	-
GlycA	-		0.67	<0.001
Glucose, mmol/L	0.17	<0.001	0.17	<0.001
Total cholesterol, mmol/L	0.17	<0.001	0.13	<0.001
Non-HDL cholesterol, mmol/L	0.21	<0.001	0.17	<0.001
HDL cholesterol, mmol/L	-0.13	<0.001	-0.17	<0.001
Triglycerides, mmol/L	0.26	<0.001	0.21	<0.001
eGFRcrea-cysC (ml/min per 1.73 m^2^)	-0.23	<0.001	-0.25	<0.001
UAE, mg/24h	0.18	<0.001	0.17	<0.001

Data are presented as standardized B coefficient (std B) with corresponding P-value. Abbreviations: *BMI*, body mass index*; eGFRcrea-cysC*, estimated glomerular filtration rate based on creatinine-cystatin C equation; *DBP*, diastolic blood pressure; *SBP*, systolic blood pressure; *HDL-cholesterol*, high density lipoprotein cholesterol; *hsCRP*; high sensitive- C-reactive protein; *UAE*, urinary albumin excretion.

**Fig 2:**
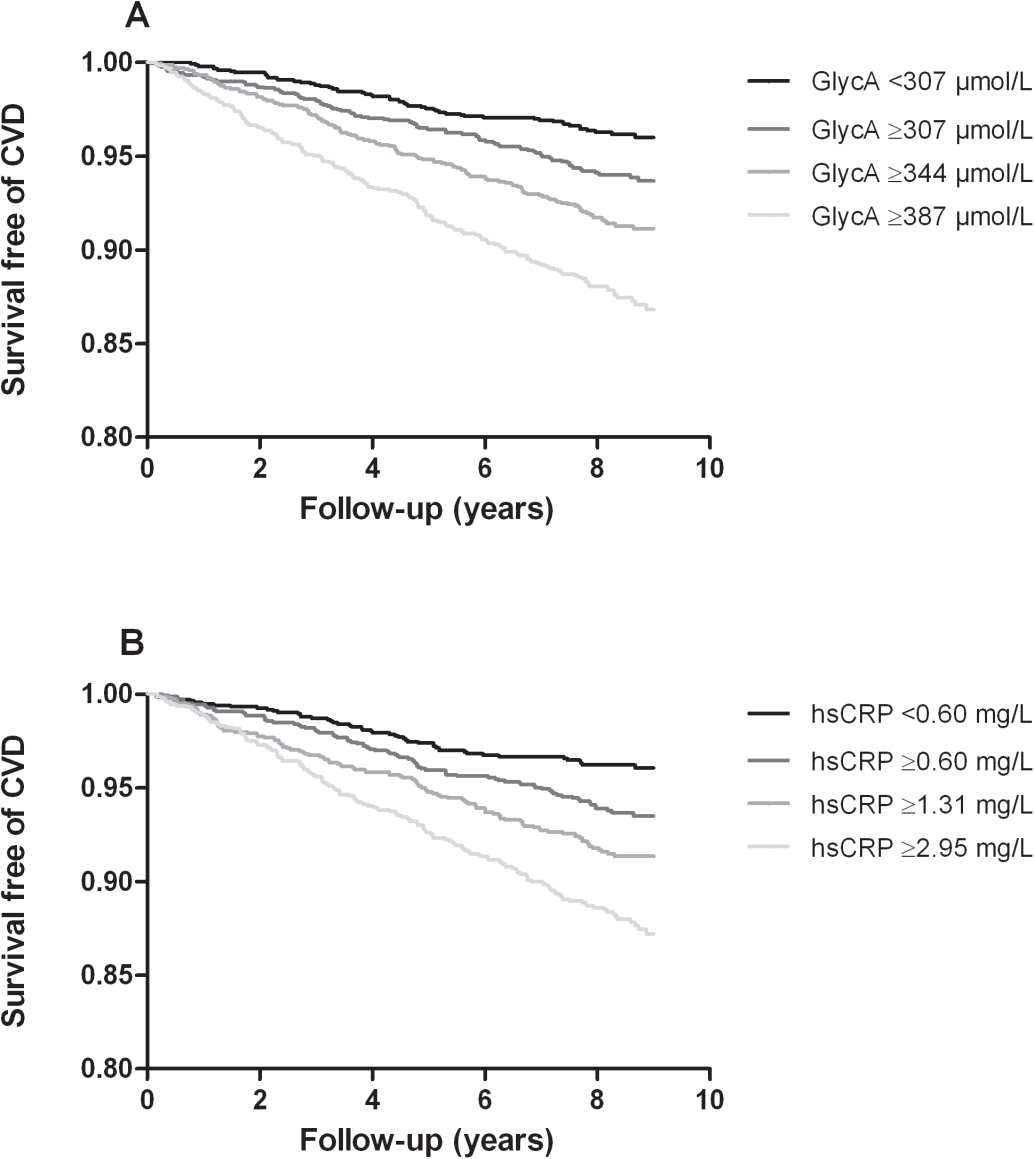
Kaplan-Meier curves showing incident cardiovascular events according to GlycA, *P*≤0.001 by log-rank test (A) and high sensitivity C-reactive protein (hsCRP), *P*≤0.001 by log-rank test (B).

After a median follow-up period of 8.5 (7.9–9.0) years 298 CVD events occurred (6.3%); 210 (70.5%) of these events were cardiac. Kaplan Meier curves for GlycA and hsCRP are shown in **Fig 2**. The Schoenfeld residuals did not suggest deviations from proportionality, supporting the assumption of proportional hazards. Associations of GlycA and hsCRP with CVD risk are shown in **Table 3**. In crude analysis, there was a robust dose response effect of GlycA on incident CVD (P for trend<0.001). Results remained essentially similar after additional adjustment for age and sex (model 1). Further adjustment for BMI, alcohol intake, smoking status (model 2), as well as T2DM, SBP, lipid lowering drug, anti-hypertensive medications (model 3) and lipid levels (model 4) did not result in any substantial change. Additional adjustment for eGFR and UAE (model 5) did not alter the results. Although the association of GlycA with incident CVD when evaluated as P for trend was no longer significant after adjustment for hsCRP, GlycA remained independently associated with CVD when evaluated per SD change (model 6). Results for CRP were comparable to those for GlycA. As with GlycA the association of hsCRP with incident CVD remained present taking account of eGFR and UAE (model 5). Also, when evaluated per SD change the association of hsCRP with incident CVD was still significant after adjustment for GlycA (model 6). There were no statistically significant interactions between GlycA or hsCRP and age or sex on CVD outcome [interactions: P>0.10 for all, data not shown].

**Table 3:** Association between GlycA and hsCRP levels and cardiovascular event in 4,759 participants (298 events) of the PREVEND study.

	Quartile 1	Quartile 2	P-value	Quartile 3	P-value	Quartile 4	P-value	P for trend*	Per 1 SD**	P-value
**GlycA**										
Participants (n)	1163	1213		1193		1190				
Range, μmol/L	<307	≥307–343		≥344–386		≥387				
No. of cases (%)	35 (3.0)	58 (4.8)		85 (7.1)		120 (10.1)				
Person years	9342	9652		9393		8988				
Crude	(reference)	1.60 [1.05–2.44]	0.03	2.41 [1.63–3.58]	<0.001	3.56 [2.44–5.19]	<0.001	<0.001	1.53 [1.39–1.67]	<0.001
Multivariable model 1	(reference)	1.27 [0.83–1.94]	0.26	1.91 [1.28–2.84]	0.001	2.75 [1.88–4.04]	<0.001	<0.001	1.41 [1.29–1.54]	<0.001
Multivariable model 2	(reference)	1.16 [0.76–1.78]	0.50	1.60 [1.06–2.40]	0.02	2.09 [1.40–3.13]	<0.001	<0.001	1.34 [1.21–1.48]	<0.001
Multivariable model 3	(reference)	1.09 [0.71–1.68]	0.68	1.43 [0.95–2.15]	0.09	1.84 [1.22–2.76]	0.003	<0.001	1.28 [1.16–1.42]	<0.001
Multivariable model 4	(reference)	1.02 [0.66–1.57]	0.94	1.29 [0.85–1.95]	0.23	1.58 [1.05–2.37]	0.03	0.004	1.24 [1.12–1.38]	<0.001
Multivariable model 5	(reference)	1.04 [0.66–1.64]	0.88	1.41 [0.91–2.19]	0.12	1.75 [1.13–2.70]	0.01	0.001	1.27 [1.14–1.42]	<0.001
Multivariable model 6	(reference)	0.94 [0.59–1.50]	0.80	1.19 [0.76–1.87]	0.45	1.31 [0.81–2.12]	0.27	0.18	1.16 [1.01–1.33]	0.04
**hsCRP**										
Participants (n)	1187	1190		1191		1191				
Range, mg/L	<0.60	≥0.60–1.30		≥1.31–2.94		≥2.95				
No. of cases (%)	37 (3.1)	54 (4.5)		83 (7.0)		124 (10.4)				
Person years	9507	9485		9333		9050				
Crude	(reference)	1.46 [0.96–2.22]	0.08	2.28 [1.55–3.36]	<0.001	3.52 [2.44–5.08]	<0.001	<0.001	1.65 [1.48–1.84]	<0.001
Multivariable model 1	(reference)	1.05 [0.69–1.60]	0.82	1.43 [0.96–2.11]	0.08	2.22 [1.53–3.23]	<0.001	<0.001	1.47 [1.31–1.66]	<0.001
Multivariable model 2	(reference)	0.96 [0.63–1.48]	0.87	1.13 [0.75–1.71]	0.55	1.69 [1.13–2.51]	0.01	<0.001	1.36 [1.19–1.54]	<0.001
Multivariable model 3	(reference)	0.91 [0.59–1.40]	0.67	1.06 [0.70–1.60]	0.79	1.53 [1.02–2.28]	0.04	0.001	1.33 [1.16–1.51]	<0.001
Multivariable model 4	(reference)	0.84 [0.55–1.29]	0.43	0.95 [0.63–1.44]	0.82	1.34 [0.89–2.01]	0.16	0.003	1.28 [1.12–1.47]	<0.001
Multivariable model 5	(reference)	0.93 [0.59–1.47]	0.75	1.11 [0.71–1.72]	0.66	1.55 [1.01–2.40]	0.05	0.001	1.34 [1.16–1.54]	<0.001
Multivariable model 6	(reference)	0.87 [0.55–1.37]	0.87	0.97 [0.62–1.51]	0.88	1.19 [0.75–1.90]	0.46	0.10	1.20 [1.01–1.43]	0.04

Hazard ratios were derived from Cox proportional hazards regression models.

Multivariable model 1: crude model + age, sex

Multivariable model 2: model 1 + BMI, alcohol intake, smoking status

Multivariable model 3: model 2 + type 2 diabetes mellitus, systolic blood pressure, lipid lowering drugs and anti-hypertensive medications

Multivariable model 4: model 3 + total cholesterol, HDL cholesterol, triglycerides.

Multivariable model 5: model 4 + eGFRcrea-cystatin C, UAE

Multivariable model 6: model 5 + hsCRP (for GlycA analyses) + GlycA (for hsCRP analyses).

Triglycerides, UAE and hsCRP were log transformed when used as a continuous variable in the analyses.

*Tests of trend across increasing quartiles were conducted by assigning the median for each quartile as its value and treating this as a continuous variable.

** 1 SD is 60.4 μmol/L for GlycA and 1.1 mg/L for hsCRP (hsCRP was natural log transformed). Abbreviations: *BMI*, body mass index*; eGFRcrea-cysC*, estimated glomerular filtration rate based on creatinine-cystatin C equation; *HDL-cholesterol*, high density lipoprotein cholesterol; *hsCRP*, high-sensitivity C-reactive protein; *UAE*, urinary albumin excretion.

When the analyses were restricted to the cardiac domain (210 events) GlycA was associated with incident cardiac events in analyses adjusted for clinical covariates, lipids, eGFR and UAE (**S1 Table**), both when evaluated as P for trend (HR 1.72 (95% CI, 1.02–2.89), P = 0.009) and as per SD change (HR 1.23 (95% CI, 1.07–1.40), P = 0.003). In analysis in which we additionally adjusted for hsCRP (cf. model 6, Table 3), GlycA was still associated with incident cardiac events (HR 1.19 (95% CI, 1.01–1.42), P = 0.04), whereas the fully adjusted association of hsCRP with cardiac events was not significant (HR 1.05 (95% CI, 0.87–1.26), P = 0.62).

The Harrell’s C-index for the Cox regression model with age and sex was 0.77 (95% CI: 0.75–0.80), indicating that 77% of the patients were correctly classified. Addition of GlycA or hsCRP to this basic model resulted in a significant improvement in the C-statistic (0.016 (95% CI, 0.006–0.02), P = 0.001 and 0.012 (95% CI, 0.003–0.022), P = 0.01, respectively). However, on top of a Cox regression model which included age, sex, BMI, smoking status and alcohol use, addition of neither GlycA, nor hsCRP resulted in a significant change in the C-statistic (0.005 (95% CI: -0.001–0.012), P = 0.10 and 0.003 (95% CI, -0.003–0.009), P = 0.34).

**Fig 3:**
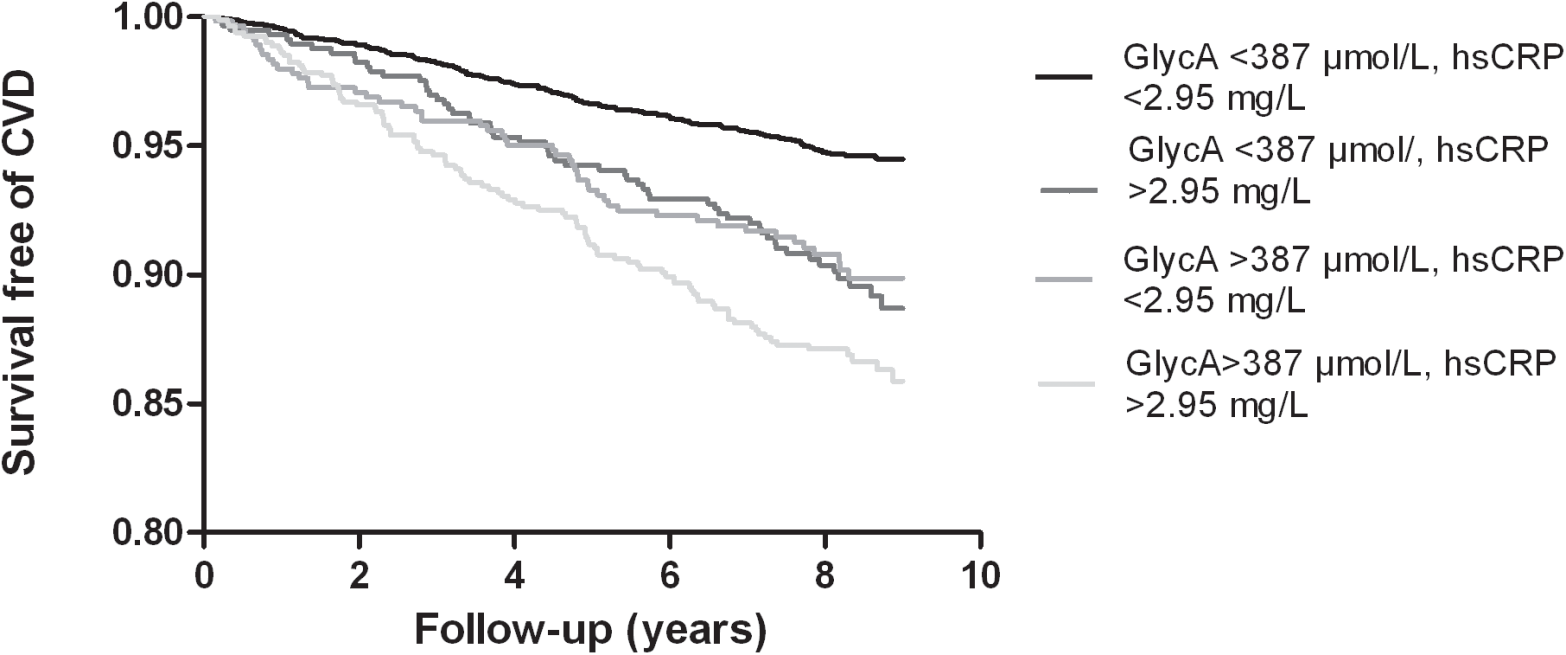
Kaplan-Meier curves of incident cardiovascular events according to joint levels of higher GlycA and hsCRP, *P*≤0.001 by log-rank test. Higher levels of GlycA and hsCRP were defined as >387 μmol/L for GlycA and >2.95 mg/L for hsCR in both sexes combined. *hsCRP*, high sensitivity C-reactive protein.

We next performed a joint analysis based on dichotomized subgroups with higher and lower GlycA or hsCRP in both sexes combined. Kaplan-Meier curves for the 4 prespecified groups of high and low GlycA or hsCRP are shown in **Fig 3**. In crude as well as in age-and sex-adjusted analysis higher GlycA (>387 μmol/L) and higher hsCRP (>2.95 mg/L) both alone and combined were associated with increased CVD risk compared with the reference group of subjects with lower GlycA and lower hsCRP (**Table 4**). After multivariable adjustment, simultaneous occurrence of higher hsCRP and higher GlycA (GlycA > 387 μmol/L and hsCRP >2.95 mg/L) was still associated increased CVD risk, whereas the association of either higher GlycA or higher hsCRP with incident CVD had lost significance (**Table 4**).

**Table 4:** Associations of joint GlycA and hsCRP levels and cardiovascular events in 4,759 participants (298 events) of the PREVEND study.

	Low GlycA and low hsCRP	Low GlycA and high hsCRP	P-value	High GlycA and low hsCRP	P-value	High GlycA and high hsCRP	P-value
Participants (n)	3079	503		479		688	
No. of cases (%)	135 (4.4)	45 (8.9)		39 (8.1)		79 (11.5)	
Crude	(reference)	2.09 [1.49–2.93]	<0.001	1.93 [1.35–2.75]	<0.001	2.81 [2.13–3.71]	<0.001
Model 1	(reference)	1.64 [1.17–2.30]	0.004	1.68 [1.18–2.40]	0.004	2.41 [1.82–3.20]	<0.001
Model 2	(reference)	1.38 [0.96–1.98]	0.08	1.27 [0.87–1.85]	0.22	1.79 [1.31–2.46]	<0.001

Higher levels were defined as greater than the upper quartile, GlycA >387 μmol/L and hsCRP >2.95 mg/L.

Multivariable model 1: Crude + age, sex.

Multivariable model 2: Model 1 + BMI, alcohol intake, smoking status, diabetes mellitus type 2, lipid lowering drugs, anti-hypertensive medications and systolic blood pressure, total cholesterol, HDL cholesterol, triglycerides, eGFRcrea-cysC and UAE.

Triglycerides, UAE and hsCRP were log transformed when used as a continuous variable in the analyses.

Abbreviations: *BMI*, body mass index*; HDL-cholesterol*, high density lipoprotein cholesterol; eGFR*crea-cysC*, estimated glomerular filtration rate based on creatinine-cystatin C equation; *hsCRP*, high-sensitivity C-reactive protein; *UAE*, urinary albumin excretion.

In secondary analysis, in which we accounted for the design of the PREVEND study, the association between GlycA and hsCRP and CVD events was still significant after adjustment for clinical covariates, lipids and eGFR (**S2 Table**). Furthermore, after adjustment for hsCRP, the association between GlycA and CVD events remained significant when analyzed per 1 SD change (HR 1.16 (95% CI, 1.01–1.34), P = 0.04); however, the P for trend was no longer statistically significant (P for trend = 0.10). Likewise, the fully adjusted association of hsCRP with CVD events was borderline significant when expressed per SD change (HR 1.16 (95% CI: 1.00–1.35), P = 0.05).

## Discussion

This prospective study among 4,759 men and women demonstrates that a recently developed high throughput NMR spectroscopy-based glycoprotein biomarker, designated GlycA, is associated with incident CVD. This association was not significantly different in men and women, and was independent of clinical risk factors and plasma lipids. Moreover, this association was not appreciably attenuated after further adjustment for eGFR and albuminuria. Of note, the extent to which GlycA predicted CVD risk was comparable to that for hsCRP. When these pro-inflammatory biomarkers were included together in the analyses, their associations with incident CVD were attenuated. Furthermore, CVD risk was highest in subjects with simultaneously higher GlycA and hsCRP, which suggests that these biomarkers may have additive potential in predicting CVD risk. Taken together, the present findings underscore the proposed role of altered protein glycan dynamics in inflammatory processes that may conceivably play a role in the development of atherosclerotic CVD.

hsCRP is a single biomarker of low-grade systemic inflammation. In contrast, the GlycA assay represents an NMR signal derived from residues within the carbohydrate side-chains of several of the most important acute-phase proteins [11,12,25]. It should be noted that the GlycA assay is not designed for determination of the relative contributions of the individually captured glycosylated protein moieties to the NMR signal. Interesting though, one of the acute phase proteins included in the NMR signal is α1-acid glycoprotein, which has been shown to be an independent predictor of cardiovascular morbidity and mortality in other large cohorts [26,27]. A potential advantage of the GlycA measurement is that its level is less variable than that of hsCRP [11]. Glycan structures of acute-phase proteins are modified under chronic inflammatory conditions [1,11]. The robust positive correlation of GlycA with hsCRP, as reinforced here, supports the contention that this glycoprotein biomarker reflects a pro-inflammatory state [11,12]. The positive relationships between GlycA and CVD risk factors, such as obesity, smoking, hypertension as well as total cholesterol, non-HDL cholesterol and triglycerides, and its inverse correlation with HDL cholesterol are in full agreement with recent reports [11,13,28]. Moreover, GlycA was higher in T2DM subjects, although it appears that the extent to which GlycA varies according to glucose tolerance status is modest [28]. Very similar relationships of these variables were found for hsCRP. The proposed pathogenic role of low-grade systemic inflammation in renal function loss, and the association of hsCRP with albuminuria [17,18,29–32] led us to determine the relationships of GlycA with eGFR and albuminuria. Of further relevance, acute-phase proteins, such as α1-acid glycoprotein and α1-antitrypsin which are captured by the GlycA assay, are present in human urine [33,34]. α1-Acid glycoprotein is correlated with albuminuria, and its urinary excretion is increased in diabetic nephropathy [34]. In hemodialysis patients, circulating levels of α1-acid glycoprotein are inversely associated with serum albumin, which in turn may predicts mortality [35]. A potentially relevant novel finding of our study is that GlycA, like hsCRP, was inversely associated with eGFR and positively with albuminuria. Given the relevance of even mild degrees of chronic kidney disease and albuminuria for the development of atherosclerotic CVD [14,15] and the presently observed relationship of GlycA with renal function abnormalities, it is remarkable that the association of GlycA with incident CVD was not attenuated after adjustment for eGFR and albuminuria. This observation raises the possibility that the impact of alterations in glycoprotein metabolism and of renal functional changes on CVD may at least in part be attributable to distinct biological pathways.

It is evident that our central finding that GlycA predicts incident CVD independent of clinical and laboratory covariates agrees with recent results from the Women’s Health Study [13]. We were also able to demonstrate that GlycA improved CVD risk classification when added to a simple model including age and sex. However, the extent to which adding GlycA improved CVD risk classification did not reach significance in analysis taking account of established CVD risk factors. Thus, it is anticipated that results from additional case-cohort studies are required to more robustly discern whether GlycA may independently improve prediction of new onset CVD.

Several methodological aspects of our study should be addressed. We consider the comprehensive assessment of laboratory variables, including lipoprotein fractions, eGFR and albuminuria in a large population of men and women a strength of our study. Second, as in previous reports [6,13,36,37], we only included subjects without clinically manifest cardiovascular disease at baseline. Thus the association of GlycA with recurrent CVD remains to be established. Third, the PREVEND study population consists of almost only Caucasians. Therefore, the applicability of the current results to other ethnicities remains uncertain. Fourth, subjects with elevated urinary albumin excretion are overrepresented in the PREVEND cohort. Of note, the association of GlycA with incident CVD remained present after adjustment for eGFR and albuminuria, and a secondary analysis taking account of the design of the PREVEND study showed similar results. Nonetheless, we cannot completely rule out the possibility of residual confounding. Finally, it should be emphasized that the process of glycosylation should be discerned from protein glycation, whereby a sugar molecule is covalently bound to a protein via a non-enzymatic mechanism.

In conclusion, this prospective cohort study involving both men and women demonstrates that GlycA, a new pro-inflammatory glycoprotein biomarker is associated with future CVD events, independent of clinical and laboratory variables including renal function. The association of GlycA with incident CVD is as strong as that of hsCRP. CVD risk was highest in the context of simultaneously higher GlycA and hsCRP, suggesting that these biomarkers may have additive potential in predicting CVD.

## Supporting Information

S1 Table Association between GlycA and hsCRP levels and cardiac events in 4,759 participants (210 events) of the PREVEND study. Multivariable model 1: crude model + age, sex. Multivariable model 2: model 1 + BMI, alcohol intake, smoking status. Multivariable model 3: model 2 + diabetes, lipid lowering drugs, anti-hypertensive medications and systolic blood pressure. Multivariable model 4: model 3 + total colesterol, HDL colesterol, triglycerides. Multivariable model 5: model 4 + eGFRcrea-cystatin C, UAE. Multivariable model 6: model 5 + hsCRP (for GlycA analyses) + GlycA (for hsCRP analyses). Triglycerides, UAE and hsCRP were log transformed when used as a continuous variable in the analyses. *Tests of trend across increasing quartiles were conducted by assigning the median for each quartile as its value and treating this as a continuous variable. ** 1 SD is 60.4 μmol/L for GlycA and 1.1 mg/L for hsCRP (hsCRP was natural log transformed). Abbreviations: *BMI*, body mass index*; eGFRcrea-cysC*, estimated glomerular filtration rate based on creatinine-cystatin C equation; *HDL-cholesterol*, high density lipoprotein cholesterol; *hsCRP*, high–sensitivity C-reactive protein; *UAE*, urinary albumin excretion.
(DOCX)

S2 Table Association between GlycA and hsCRP levels and cardiovascular events in 4,759 participants (298 events) of the PREVEND study, accounting for the sampling design of the study (presence or absence of a urinary albumin concentration >10 mg/L) by specifying stratum-specific baseline hazard functions. Multivariable model 1: crude + age and sex. Multivariable model 2: model 1 + BMI, alcohol intake, smoking status, diabetes, lipid lowering drugs, anti-hypertensive medications, systolic blood pressure, total cholesterol, HDL cholesterol, triglycerides, eGFRcrea-cystatin C. Multivariable model 3: model 2 + hsCRP (for GlycA analyses) + GlycA (for hsCRP anlyses). Triglycerides and hsCRP were log transformed when used as a continuous variable in the analyses. *Tests of trend across increasing quartiles were conducted by assigning the median for each quartile as its value and treating this as a continuous variable. ** 1 SD is 60.4 μmol/L for GlycA and 1.1 mg/L for hsCRP (hsCRP was natural log transformed). Abbreviations: *BMI*, body mass index*; eGFRcrea-cysC*, estimated glomerular filtration rate based on creatinine-cystatin C equation; *HDL-cholesterol*, high density lipoprotein cholesterol; *hsCRP*, high–sensitivity C-reactive protein; *UAE*, urinary albumin excretion.
(DOCX)
