# Supplementary material for: GlycA, a Pro-Inflammatory Glycoprotein Biomarker, and Incident Cardiovascular Disease: Relationship with C-Reactive Protein and Renal Function
Source: PLoS One. 2015 Sep 23;10(9):e0139057. doi: 10.1371/journal.pone.0139057 (PMC4580603; doi:10.1371/journal.pone.0139057)
Supplement: S1 Table — Multivariable model 1: crude model + age, sex. Multivariable model 2: model 1 + BMI, alcohol intake, smoking status. Multivariable model 3: model 2 + diabetes, lipid lowering drugs, anti-hypertensive medications and systolic blood pressure. Multivariable model 4: model 3 + total colesterol, HDL colesterol, triglycerides. Multivariable model 5: model 4 + eGFRcrea-cystatin C, UAE. Multivariable model 6: model 5 + hsCRP (for GlycA analyses) + GlycA (for hsCRP analyses). Triglycerides, UAE and hsCRP were log transformed when used as a continuous variable in the analyses. *Tests of trend across increasing quartiles were conducted by assigning the median for each quartile as its value and treating this as a continuous variable. ** 1 SD is 60.4 μmol/L for GlycA and 1.1 mg/L for hsCRP (hsCRP was natural log transformed). Abbreviations: BMI, body mass index; eGFRcrea-cysC, estimated glomerular filtration rate based on creatinine-cystatin C equation; HDL-cholesterol, high density lipoprotein cholesterol; hsCRP, high–sensitivity C-reactive protein; UAE, urinary albumin excretion. (DOCX) [file pone.0139057.s001.docx]

|  | | **Quartile 1** | **Quartile 2** | P-value | **Quartile 3** | P-value | **Quartile 4** | P-value | P for trend* | Per SD** | P-value |
| --- | --- | --- | --- | --- | --- | --- | --- | --- | --- | --- | --- |
| **GlycA** |  |  |  |  |  |  |  |  |  |  |  |
| Participants (n) | | 1163 | 1213 |  | 1193 |  | 1190 |  |  |  |  |
| Range, µmol/L | | <307 | ≥307-343 |  | ≥344-386 |  | ≥387 |  |  |  |  |
| No. of cases (%) | | 25 (2.1) | 44 (3.6) |  | 59 (4.9) |  | 82 (6.9) |  |  |  |  |
| Person years | | 9342 | 9652 |  | 9393 |  | 8988 |  |  |  |  |
| Crude | | (reference) | 1.70 [1.04-2.78] | 0.03 | 2.35 [1.47-3.75] | <0.001 | 3.41 [2.18-5.34] | <0.001 | <0.001 | 1.47 [1.32-1.65] | <0.001 |
| Multivariable model 1 | | (reference) | 1.39 [0.85-2.27] | 0.19 | 1.95 [1.22-3.13] | 0.006 | 2.80 [1.78-4.42] | <0.001 | <0.001 | 1.38 [1.24-1.54] | <0.001 |
| Multivariable model 2 | | (reference) | 1.27 [0.77-2.10] | 0.35 | 1.64 [1.01-2.67] | 0.05 | 2.16 [1.34-3.49] | 0.002 | <0.001 | 1.30 [1.15-1.47] | <0.001 |
| Multivariable model 3 | | (reference) | 1.21 [0.73-2.00] | 0.47 | 1.47 [0.90-2.41] | 0.12 | 1.90 [1.17-3.09] | 0.009 | 0.002 | 1.25 [1.10-1.41] | 0.001 |
| Multivariable model 4 | | (reference) | 1.10 [0.67-1.83] | 0.70 | 1.29 [0.79-2.11] | 0.31 | 1.55 [0.95-2.52] | 0.08 | 0.04 | 1.19 [1.04-1.36] | 0.01 |
| Multivariable model 5 | | (reference) | 1.07 [0.62-1.85] | 0.80 | 1.43 [0.85-2.40] | 0.18 | 1.72 [1.02-2.89] | 0.04 | 0.009 | 1.23 [1.07-1.40] | 0.003 |
| Multivariable model 6 | | (reference) | 1.03 [0.60-1.79] | 0.91 | 1.33 [0.78-2.29] | 0.30 | 1.54 [0.87-2.73] | 0.14 | 0.11 | 1.19 [1.01-1.42] | 0.04 |
| **hsCRP** |  |  |  |  |  |  |  |  |  |  |  |
| Participants (n) | | 1187 | 1190 |  | 1191 |  | 1191 |  |  |  |  |
| Range, mg/L | | <0.60 | ≥0.60-1.30 |  | ≥1.31-2.94 |  | ≥2.95 |  |  |  |  |
| No. Of ‘cases’ (%) | | 30 (2.5) | 42 (3.5) |  | 59 (5.0) |  | 79 (6.6) |  |  |  |  |
| Person years | | 9507 | 9485 |  | 9333 |  | 9050 |  |  |  |  |
| Crude | | (reference) | 1.40 [0.88-2.24] | 0.16 | 2.00 [1.29-3.11] | 0.002 | 2.77 [1.82-4.21] | <0.001 | <0.001 | 1.51 [1.33-1.73] | <0.001 |
| Multivariable model 1 | | (reference) | 1.03 [0.64-1.64] | 0.92 | 1.29 [0.83-2.02] | 0.26 | 1.82 [1.18-2.80] | 0.006 | <0.001 | 1.36 [1.18-1.57] | <0.001 |
| Multivariable model 2 | | (reference) | 0.94 [0.58-1.52] | 0.80 | 1.01 [0.63-1.61] | 0.97 | 1.35 [0.85-2.13] | 0.20 | 0.04 | 1.23 [1.05-1.44] | 0.01 |
| Multivariable model 3 | | (reference) | 0.88 [0.54-1.42] | 0.60 | 0.95 [0.59-1.51] | 0.81 | 1.22 [0.77-1.94] | 0.40 | 0.08 | 1.20 [1.02-1.41] | 0.02 |
| Multivariable model 4 | | (reference) | 0.79 [0.49-1.28] | 0.33 | 0.81 [0.51-1.30] | 0.39 | 1.01 [0.63-1.61] | 0.97 | 0.28 | 1.13 [0.96-1.34] | 0.13 |
| Multivariable model 5 | | (reference) | 0.91 [0.54-1.53] | 0.71 | 0.97 [0.59-1.61] | 0.92 | 1.22 [0.74-2.03] | 0.43 | 0.13 | 1.20 [1.01-1.42] | 0.04 |
| Multivariable model 6 | | (reference) | 0.85 [050-1.43] | 0.53 | 0.85 [0.51-1.42] | 0.53 | 0.93 [0.54-1.61] | 0.80 | 0.97 | 1.05 [0.86-1.30] | 0.62 |
