# Supplementary material for: GlycA, a Pro-Inflammatory Glycoprotein Biomarker, and Incident Cardiovascular Disease: Relationship with C-Reactive Protein and Renal Function
Source: PLoS One. 2015 Sep 23;10(9):e0139057. doi: 10.1371/journal.pone.0139057 (PMC4580603; doi:10.1371/journal.pone.0139057)
Supplement: S2 Table — Multivariable model 1: crude + age and sex. Multivariable model 2: model 1 + BMI, alcohol intake, smoking status, diabetes, lipid lowering drugs, anti-hypertensive medications, systolic blood pressure, total cholesterol, HDL cholesterol, triglycerides, eGFRcrea-cystatin C. Multivariable model 3: model 2 + hsCRP (for GlycA analyses) + GlycA (for hsCRP anlyses). Triglycerides and hsCRP were log transformed when used as a continuous variable in the analyses. *Tests of trend across increasing quartiles were conducted by assigning the median for each quartile as its value and treating this as a continuous variable. ** 1 SD is 60.4 μmol/L for GlycA and 1.1 mg/L for hsCRP (hsCRP was natural log transformed). Abbreviations: BMI, body mass index; eGFRcrea-cysC, estimated glomerular filtration rate based on creatinine-cystatin C equation; HDL-cholesterol, high density lipoprotein cholesterol; hsCRP, high–sensitivity C-reactive protein; UAE, urinary albumin excretion. (DOCX) [file pone.0139057.s002.docx]

|  | | **Quartile 1** | **Quartile 2** | P-value | **Quartile 3** | P-value | **Quartile 4** | P-value | P for trend* | Per SD** | P-value |
| --- | --- | --- | --- | --- | --- | --- | --- | --- | --- | --- | --- |
| **GlycA** |  |  |  |  |  |  |  |  |  |  |  |
| Participants (n) | | 1163 | 1213 |  | 1193 |  | 1190 |  |  |  |  |
| Range, µmol/L | | <307 | ≥307-343 |  | ≥344-386 |  | ≥387 |  |  |  |  |
| No. of cases (%) | | 35 (3.0) | 58 (4.8) |  | 85 (7.1) |  | 120 (10.1) |  |  |  |  |
| Person years | | 9342 | 9652 |  | 9393 |  | 8988 |  |  |  |  |
| Crude | | (reference) | 1.60 [1.05-2.43] | 0.03 | 2.39 [1.61-3.54] | <0.001 | 3.49 [2.39-5.08] | <0.001 | <0.001 | 1.51 [1.38-1.66] | <0.001 |
| Multivariable model 1 | | (reference) | 1.27 [0.83-1.93] | 0.28 | 1.87 [1.25-2.78] | 0.002 | 2.66 [1.81-3.91] | <0.001 | <0.001 | 1.40 [1.28-1.53] | <0.001 |
| Multivariable model 2 | | (reference) | 1.04 [0.66-1.64] | 0.87 | 1.43 [0.93-2.22] | 0.11 | 1.75 [1.14-2.71] | 0.01 | 0.001 | 1.27 [1.14-1.42] | <0.001 |
| Multivariable model 3 | | (reference) | 0.95 [0.60-1.51] | 0.82 | 1.21 [0.77-1.91] | 0.40 | 1.33 [0.82-2.15] | 0.24 | 0.10 | 1.16 [1.01-1.34] | 0.04 |
| **hsCRP** |  |  |  |  |  |  |  |  |  |  |  |
| Participants (n) | | 1187 | 1190 |  | 1191 |  | 1191 |  |  |  |  |
| Range, mg/L | | <0.60 | ≥0.60-1.30 |  | ≥1.31-2.94 |  | ≥2.95 |  |  |  |  |
| No. of cases (%) | | 37 (3.1) | 54 (4.5) |  | 83 (7.0) |  | 124 (10.4) |  |  |  |  |
| Person years | | 9507 | 9485 |  | 9333 |  | 9050 |  |  |  |  |
| Crude | | (reference) | 1.45 [0.95-2.20] | 0.08 | 2.29 [1.55-3.37] | <0.001 | 3.47 [2.41-5.01] | <0.001 | <0.001 | 1.64 [1.47-1.83] | <0.001 |
| Multivariable model 1 | | (reference) | 1.04 [0.68-1.58] | 0.87 | 1.41 [0.95-2.10] | 0.09 | 2.16 [1.49-3.15] | <0.001 | <0.001 | 1.46 [1.29-1.64] | <0.001 |
| Multivariable model 2 | | (reference) | 0.90 [0.57-1.41] | 0.64 | 1.07 [0.69-1.65] | 0.76 | 1.51 [0.98-2.31] | 0.06 | 0.001 | 1.33 [1.16-1.53] | <0.001 |
| Multivariable model 3 | | (reference) | 0.84 [0.53-1.32] | 0.44 | 0.93 [0.60-1.45] | 0.76 | 1.15 [0.72-1.83] | 0.55 | 0.11 | 1.19 [1.00-1.42] | 0.05 |
